# Improved effectiveness of stereotactic radiosurgery in large brain metastases by individualized isotoxic dose prescription: an *in silico *study

**DOI:** 10.1007/s00066-018-1262-x

**Published:** 2018-01-18

**Authors:** Jaap D. Zindler, Jacqueline Schiffelers, Philippe Lambin, Aswin L. Hoffmann

**Affiliations:** 1 Department of Radiation Oncology (MAASTRO), GROW School for Oncology and Developmental Biology, Maastricht University Medical Center, Dr. Tanslaan 12, 6229 ET Maastricht, The Netherlands; 2 Institute of Radiooncology-OncoRay, Helmholtz-Zentrum Dresden-Rossendorf, Bautzener Landstraße 400, 01328 Dresden, Germany; 3 Department of Radiation Oncology, Faculty of Medicine and University Hospital Carl Gustav Carus, Technische Universität Dresden, Fetscherstraße 74, 01307 Dresden, Germany

**Keywords:** Radiotherapy, Stereotactic, Dose prescription, Normal tissue tolerance, Large brain metastases, Strahlentherapie, Stereotaktisch, Dosisverschreibung, Normalgewebetoleranz, Große Hirnmetastasen

## Abstract

**Introduction:**

In large brain metastases (BM) with a diameter of more than 2 cm there is an increased risk of radionecrosis (RN) with standard stereotactic radiosurgery (SRS) dose prescription, while the normal tissue constraint is exceeded. The tumor control probability (TCP) with a single dose of 15 Gy is only 42%. This *in silico *study tests the hypothesis that isotoxic dose prescription (IDP) can increase the therapeutic ratio (TCP/Risk of RN) of SRS in large BM.

**Materials and methods:**

A treatment-planning study with 8 perfectly spherical and 46 clinically realistic gross tumor volumes (GTV) was conducted. The effects of GTV size (0.5–4 cm diameter), set-up margins (0, 1, and 2 mm), and beam arrangements (coplanar vs non-coplanar) on the predicted TCP using IDP were assessed. For single-, three-, and five-fraction IDP dose–volume constraints of V_12Gy_ = 10 cm^3^, V_19.2_ _Gy_ = 10 cm^3^, and a V_20Gy_ = 20 cm^3^, respectively, were used to maintain a low risk of radionecrosis.

**Results:**

In BM of 4 cm in diameter, the maximum achievable single-fraction IDP dose was 14 Gy compared to 15 Gy for standard SRS dose prescription, with respective TCPs of 32 and 42%. Fractionated SRS with IDP was needed to improve the TCP. For three- and five-fraction IDP, a maximum predicted TCP of 55 and 68% was achieved respectively (non-coplanar beams and a 1 mm GTV-PTV margin).

**Conclusions:**

Using three-fraction or five-fraction IDP the predicted TCP can be increased safely to 55 and 68%, respectively, in large BM with a diameter of 4 cm with a low risk of RN. Using IDP, the therapeutic ratio of SRS in large BM can be increased compared to current SRS dose prescription.

## Zusammenfassung

**Einleitung:**

Bei einer Standarddosisverschreibung für eine stereotaktische Radiochirurgie (SRS) von großen Hirnmetastasen (BM) mit einem Durchmesser von ≥2 cm ist das Risiko für eine Strahlennekrose (RN) erhöht, da die Toleranzdosis des Hirngewebes überschritten wird. Die Tumorkontrollwahrscheinlichkeit (TCP) ist bei 15 Gy lediglich 42 %. Diese In-silico-Studie testet die Hypothese, dass eine isotoxische Dosisverschreibung (IDP) das Potenzial hat, das therapeutische Fenster (TCP/RN-Risiko) von SRS bei großen BM zu vergrößern.

**Material und Methoden:**

Eine Planungsstudie mit 8 perfekt sphärischen und 46 klinisch realistischen Tumorvolumen (GTV) wurde durchgeführt. Die Effekte der GTV-Größe (Durchmesser 0,5–4,0 cm), des Set-up-Saums (0, 1, 2 mm) und der Strahlanordnung (koplanar vs. nichtkoplanar) auf die erwartete TCP bei Anwendung der IDP wurden untersucht. Für IDP in 1, 3 oder 5 Fraktionen wurden die entsprechenden Dosis-Volumen-Einschränkungen V_12Gy_ = 10 cm^3^, V_19,2_ _Gy_ = 10 cm^3^ und V_20Gy_ = 20 cm^3^ verwendet, um das RN-Risiko gering zu halten.

**Ergebnisse:**

Für BM mit einem Durchmesser von 4 cm war die maximal erreichbare IDP-Dosis in einer Bestrahlung 14 Gy im Vergleich zu 15 Gy bei der Standarddosisverschreibung, mit entsprechender TCP von 32 % bzw. 42 %. Fraktionierte SRS mit IDP war notwendig, um die TCP zu erhöhen. Für IDP in 3 und 5 Fraktionen wurde eine maximal vorhergesagte TCP von 55 und 68 % erreicht (nichtkoplanare Strahlenbündel und GTV-PTV-Saum von 1 mm).

**Schlussfolgerung:**

Mit einer IDP in 3 oder 5 Fraktionen kann die vorhergesagte TCP bei BM mit einem Durchmesser von 4 cm jeweils auf 55 bzw. 68 % erhöht werden, mit gleichzeitig geringem Risiko auf RN. Mit IDP kann das therapeutische Fenster einer SRS großer BM im Vergleich zur Standarddosisverschreibung erhöht werden.

## Introduction

In stereotactic radiosurgery (SRS) for brain metastases (BM), the dose is generally prescribed according to a risk-adapted approach depending on the size of the planning target volume (PTV): for smaller PTVs higher SRS doses are prescribed than for larger PTVs with the aim to limit toxicity to acceptable levels in large BM [1]. In Radiation Therapy Oncology Group (RTOG) study 90-05, the maximum tolerated single-fraction dose for BM with a diameter >3 cm was 15 Gy, as a higher dose of 18 Gy was associated with an unacceptably high rate of severe central nervous system toxicity of 50% [3]. Recently, consensus was reached within the Netherlands for SRS dose prescriptions: a single dose of 24 Gy is prescribed to PTV sizes <1 cm^3^ and the dose level is stepwise decreased to 21, 18 and 15 Gy for PTV sizes between 1–10 cm^3^, between 10–20 cm^3^, and >20 cm^3^, respectively. In clinical practice, SRS is used for inoperable BM up to a diameter of 4 cm. The consequence of this PTV size-based dose prescription protocol is a 12-month local tumor control probability (TCP) of about 86% in small BM and a TCP of around 40% in large BM [4, 5]. Given the low TCP for large BM and taking into account that patients with large BM are often medically inoperable, there is a clear need for improvement of SRS in these patients, but not at the cost of an unacceptably high risk of toxicity. This is currently being investigated in phase I studies [6]. An alternative to PTV size-based dose prescription is isotoxic dose prescription (IDP) [7–13]. The quintessence of this strategy is that the normal tissue tolerance level is always respected and used as a base for dose prescription. Thereby the risk of radionecrosis is kept low. The dose in the tumor is increased to the highest dose that is technically achievable and thereby maximizing the TCP while simultaneously respecting the normal tissue constraint. The IDP concept is different from the PTV size-based dose prescription approach, where fixed prescription doses are used that solely depend on the size of the target volume and for large BM do not respect the predefined dose–volume constraint for normal tissue. From previous studies of single-fraction SRS for BM, it is known that the risk of radionecrosis increases rapidly if the volume of the surrounding healthy brain tissue receiving at least 12 Gy is greater than 10 cm^3^ (that is, V_12Gy_ > 10 cm^3^) [14–16]. Apart from being dependent on the tumor prescription dose, the V_12Gy_ also depends on the gross tumor volume-to-planning target-volume (GTV-PTV) margin used as well as on the beam arrangement (i. e., coplanar vs non-coplanar) that affect the degree of dose conformity and the steepness of the dose gradient at the outer rim of the PTV [17].

In this study, the hypothesis is tested that through IDP the predicted TCP in large BM up to 4 cm diameter can be improved from the disappointing low level of 42% that is obtained with a standard single SRS dose of 15 Gy while simultaneously respecting an acceptably low normal tissue complication probability (NTCP). Furthermore, the effects of GTV volume, different GTV-PTV margins, and beam arrangements on the predicted TCP are systematically assessed [4, 18, 19]. Both single-fraction and fractionated stereotactic radiotherapy (FSRS) IDP schemes are considered to assess the effect of fractionation.

## Materials and methods

This study comprises three phases. First, the potential to increase TCP under isotoxic conditions is investigated for single-fraction, three-fraction and five-fraction IDP schemes, with artificial BM having spherical GTV shapes of different diameters (0.5–4.0 cm, with stepwise increasing diameter of 0.5 cm, Table 1 for treatment plan characteristics). This allows us to systematically derive empirical relationships between the GTV size and the maximum achievable predicted TCP as a function of the GTV-PTV margin and the beam arrangement. Second, these results are compared against clinically delivered SRS treatment plans in 46 patients with realistically shaped GTVs.

**Table 1:** Characteristics of 48 SRS plans for artificially contoured BM

	Coplanar VMAT beam arrangement				Non-coplanar VMAT beam arrangement			
Diameter GTV/GTV -PTV margin (cm)	D_max_ (%) in PTV	D_mean_ (%) in PTV	RTOG CI	Paddick GI	D_max_ (%) in PTV	D_mean_ (%) in PTV	RTOG CI	Paddick GI
*0.5/0*	101	90	2.0	14.8	103	90	1.5	14.6
*0.5/0.1*	123	98	2.5	9.7	128	98	1.2	9.2
*0.5/0.2*	135	99	1.2	7.9	133	100	1.1	6.1
*1.0/0*	126	97	1.1	6.9	111	91	1.1	7.2
*1.0/0.1*	127	103	1.2	5.7	140	104	1.1	4.7
*1.0/0.2*	118	99	1.1	4.6	125	98	1.0	4.3
*1.5/0*	127	101	1.1	4.5	118	96	1.0	4.3
*1.5/0.1*	121	103	1.1	4.0	135	107	1.0	3.6
*1.5/0.2*	118	100	1.0	4.0	145	106	1.0	3.2
*2.0/0*	116	100	1.0	3.9	112	96	1.0	3.4
*2.0/0.1*	130	109	1.1	3.7	132	108	1.0	3.2
*2.0/0.2*	120	101	1.0	3.6	130	105	1.0	3.0
*2.5/0*	122	103	1.0	3.7	106	94	1.0	2.6
*2.5/0.1*	124	108	1.1	3.5	125	104	1.0	2.9
*2.5/0.2*	124	104	1.0	3.4	131	108	1.0	2.8
*3.0/0*	126	102	1.0	3.5	113	97	1.0	2.8
*3.0/0.1*	126	108	1.1	3.3	124	106	1.0	2.7
*3.0/0.2*	126	106	1.0	3.3	128	106	1.0	2.7
*3.5/0*	131	102	1.0	3.3	109	96	1.0	2.7
*3.5/0.1*	124	107	1.1	3.2	122	105	1.0	2.6
*3.5/0.2*	128	107	1.0	3.2	127	107	1.0	2.6
*4.0/0*	135	102	1.0	3.1	112	97	1.0	2.6
*4.0/0.1*	121	104	1.1	3.0	126	105	1.0	2.5
*4.0/0.2*	134	107	1.0	3.1	139	113	1.0	2.6

*VMAT* volumetric modulated arc therapy, *GTV* gross tumor volume, *PTV* planning target volume, *RTOG CI* Radiation Therapy Oncology Group Conformity Index, *Paddick GI* Paddick gradient index, *BM* brain metastasis, *SRS* stereotactic radiosurgery

### Nominal treatment plans: target-volume definition and treatment-planning technique

A computed tomography (CT) scan (Siemens Somatom Sensation Open, Erlangen, Germany) of an anonymized patient treated with SRS for BM was used to design nominal treatment plans having a dose prescription according to the Dutch consensus guidelines. The head and neck region was imaged until the claviculae with a slice thickness of 1 mm. In the treatment-planning system (Eclipse version 11.0.42, Varian, Palo Alto, CA, USA), 8 perfectly spherical GTVs with diameters ranging from 0.5 to 4.0 cm in steps of 0.5 cm were contoured in the right parietal lobe so that the brain stem and optic system did not overlap with the PTV. For each GTV, a PTV was created by isotropic expansion with a margin of 0, 1, and 2 mm. The dose prescription based on PTV size was a single fraction of 24, 21, 18, and 15 Gy for PTV sizes <1, 1–10, 10–20 cm^3^, and 20–65 cm^3^. With the prescribed dose, 99% of the PTV was covered, while having a steep dose gradient outside the PTV for brain sparing and allowing large dose inhomogeneity within the PTV. Per PTV, two 10 MV photon-based volumetric modulated arc therapy (VMAT, calculation grid size 1 × 1 × 1 mm^3^) plans were made by the same treatment planner, one with two overlapping coplanar arcs at a couch angle of 0° and one with three non-coplanar arcs having a couch angle of 0°, 45°, and 315° and a collimator angle of 30° or 330º. In total, 48 treatment plans were generated (Table 1). For each treatment plan, the V_12Gy_ of the healthy brain minus the GTV was determined.

### Renormalized treatment plans: IDP based on normal tissue dose constraint

For single-fraction IDP-based dose prescription, the nominal treatment plans with spherical GTVs were renormalized (by altering the monitor units) such that V_12Gy_ = 10 cm^3^ for the healthy brain minus the GTV. The corresponding IDP dose levels for each of the 48 treatment plans were assessed for further analysis. The same procedure was used for the five-fraction IDP scheme, but a V_20Gy_ = 20 cm^3^ constraint for the healthy brain minus the GTV was used instead [20]. For three-fraction IDP a V_19.2_ _Gy_ = 10 cm^3^ was used as a normal tissue constraint. This constraint was recalculated from the V_12Gy_ = 10 cm^3^ using the linear quadratic model using an α/β ratio for brain tissue of 3. The predicted TCP was calculated from the IDP prescription dose by using a dose–response model that was statistically fitted to the data points of Wiggenraad et al. [4] (see Appendix). For calculation, plotting, and rescaling of the dose–volume histograms (DVHs), in-house developed MATLAB scripts (Version 8.5; The MathWorks, Inc., Natick, MA, USA) were used.

### Validation of IDP results in clinically delivered SRS plans

Since in clinical practice the GTVs of BM are not perfectly spherical and the plan quality may slightly vary due to inter- and intratreatment planner differences, we compared the IDP dose levels obtained from the 48 treatment plans with perfectly spherical GTVs to those of clinical treatment plans comprising 46 consecutive patients who had been treated with SRS for a single BM at our institution between January 2013 and June 2014 with a dose of 15–24 Gy in 1–3 fractions. Patients had been considered eligible for SRS if they had a maximum of four BM from metastasized solid primary tumors (e. g., non-small cell lung cancer, breast cancer, colorectal cancer, melanoma, and bladder cancer) at the pretreatment contrast-enhanced magnetic resonance imaging (MRI) scan, a Karnofsky performance status of 70 or more, and extracranial treatment options. The selected cohort was identified from a database containing all patients who had been treated with SRS for newly diagnosed BM in our institution. Prior to treatment, a gadolinium contrast-enhanced MRI scan (3D T_1_-weighted sequence on a 1.5 T Ingenia/Intera or 3 T Achieva scanner, Philips Medical Systems, Eindhoven, Netherlands) was made with a slice thickness of 1 mm. Patients had been immobilized with a frameless mask and underwent an iodide contrast-enhanced CT scan (Siemens Somatom Sensation Open, Erlangen, Germany) with a slice thickness of 1 mm. For treatment-planning purposes, the MRI scan had been rigidly co-registered with the CT scan in Eclipse (Varian, Palo Alto, CA, USA). The BM had been delineated as GTV contours on the MRI scan and visually checked on the CT scan thereafter. A GTV-PTV margin of 2 mm had been applied. A VMAT (RapidArc) technique with 10 MV coplanar beams had been used to design the dose distribution for delivery with a TrueBeam STX linear accelerator (Varian, Palo Alto, CA, USA). To derive the IDP dose levels for our study, these treatment plans were renormalized such that the constraints V_12Gy_ = 10 cm^3^, V_19.2_ _Gy_ = 10 cm^3^, and V_20Gy_ = 20 cm^3^ were satisfied for the single-, three-, and five-fraction schemes, respectively.

### Statistical methods

The Pearson’s chi-squared test was used as a measure of fit between the IDP dose levels of the spherical GTVs and those of the non-spherical GTVs of the clinical plans. The difference between the coefficients of both exponential decay models was zero.

## Results

**Fig. 1:**
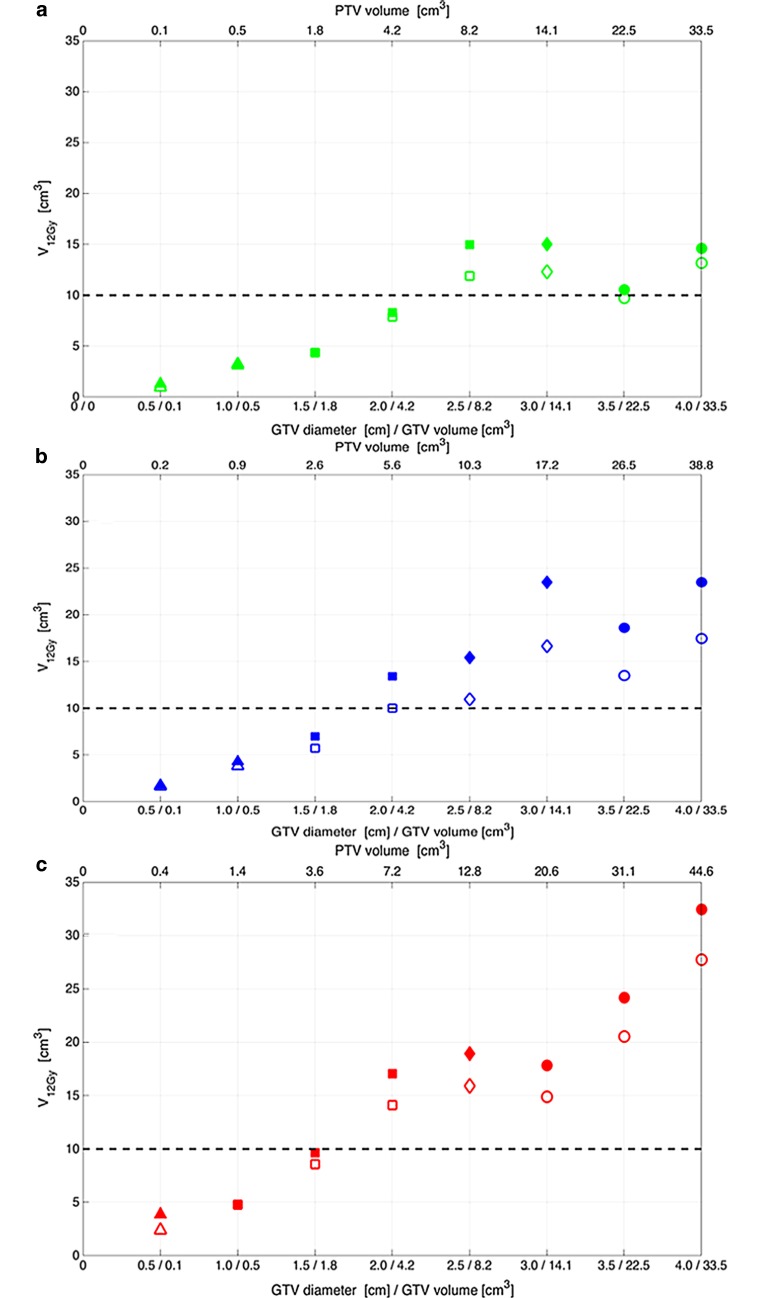
Commonly used risk-adapted dose prescription based on PTV size and its effect on exceeding the normal tissue constraint for radionecrosis risk V_12Gy_ = 10 cm^3^. The V_12Gy_ as function of GTV size is presented for different GTV-PTV margins and beam arrangements. Legend: *GTV* gross tumor volume, *PTV* planning target volume, *V*_12Gy_ volume of healthy brain tissue which is irradiated to 12 Gray or more. The GTV-PTV margin: 0 mm (**a**), 1 mm (**b**), and 2 mm (**c**). The open and filled symbols represent non-coplanar and coplanar beams, respectively. The single-fraction PTV size-based dose prescription: 24 Gy (*triangle*), 21 Gy (*square*), 18 Gy (*diamond*), and 15 Gy (*circle*)

The application of a non-zero GTV-PTV margin results in an increase in the PTV size and may hence influence the PTV size-based dose prescription and thereby the therapeutic ratio. As indicated by the change in symbol shapes in Fig. 1, applying a GTV-PTV margin of 1 mm instead of 0 mm resulted in lowering the nominal prescription dose from 21 to 18 Gy for a GTV diameter of 2.5 cm, while for the other 7 GTV diameters, the prescribed dose did not change. Applying a GTV-PTV margin of 2 mm instead of 0 mm resulted in decreasing the nominal prescription dose from 24 to 21 Gy, from 21 to 18 Gy, and from 18 to 15 Gy for GTV diameters of 1.0, 2.5, and 3.0 cm, respectively, while for the other 5 GTV diameters, the prescribed dose remained the same.

Applying a non-zero GTV-PTV margin also results in an increase of the dose absorbed in uninvolved healthy brain tissue and therefore affects the NTCP and therapeutic ratio. When the single-fraction PTV size-based dose prescription protocol is applied to the 8 perfectly spherical GTVs, V_12Gy_ shows a clear tendency to increase with increasing GTV size and increasing GTV-PTV margin and is generally lower for the non-coplanar beam arrangement than for the coplanar beams (Fig. 1). From this figure, it is evident that for GTV diameters >2 cm, V_12Gy_ exceeds the 10 cm^3^ constraint level irrespective of the GTV-PTV margin or the beam arrangement. Furthermore, this figure shows that for a GTV diameter of 4 cm, V_12Gy_ is reduced from 33 cm^3^ to 13 cm^3^ in case a GTV-PTV margin of 0 mm instead of 2 mm is used in combination with non-coplanar beams instead of coplanar beams.

**Fig. 2:**
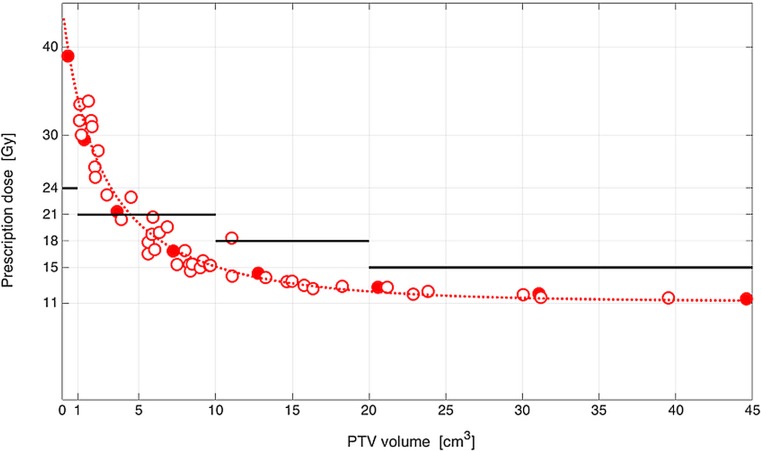
Clinical validation of plan quality of the *in silico* SRS treatment plans with artificially contoured BM compared to clinically delivered SRS treatment plans in BM patients. A comparison of the single-fraction IDP dose level with V_12Gy_ = 10 cm^3^ for coplanar beam arrangement and GTV-PTV margin of 2 mm between perfectly spherical GTVs (*filled dots*) and non-spherical GTVs of clinical plans (*open dots*). Legend: *Gy* Gray, *GTV* gross tumor volume, *PTV* planning target volume, *SRS* stereotactic radiosurgery, *BM* brain metastases, *IDP* isotoxic dose prescription, *V*_12__G__y_ volume of healthy brain tissue which is irradiated to 12 Gray or more. The *solid lines* represent single-fraction PTV size-based dose prescription protocols. The *dashed line* represents a logistic regression model fitted to data of perfectly spherical GTVs

To test whether the single-fraction IDP dose levels derived for the spherical GTVs apply to the non-spherical GTVs of the clinical treatment plans, an empirical relationship between the PTV size of the spherical GTVs and the single-fraction IDP dose level was derived by fitting an exponential decay model to the data of the coplanar beam arrangement with a GTV-PTV margin of 2 mm (Fig. 2). The same was done for the IDP dose of the 46 clinical treatment plans with non-spherical PTVs. It could be shown that there is no statistically significant difference between these fits. The median (±SD) IDP dose difference between the spherical GTVs of the artificial treatment plans and the non-spherical GTVs of the clinical treatment plans was 0.25 ± 1.70 Gy and ranged from −3.92 to 2.16 Gy.

**Fig. 3:**
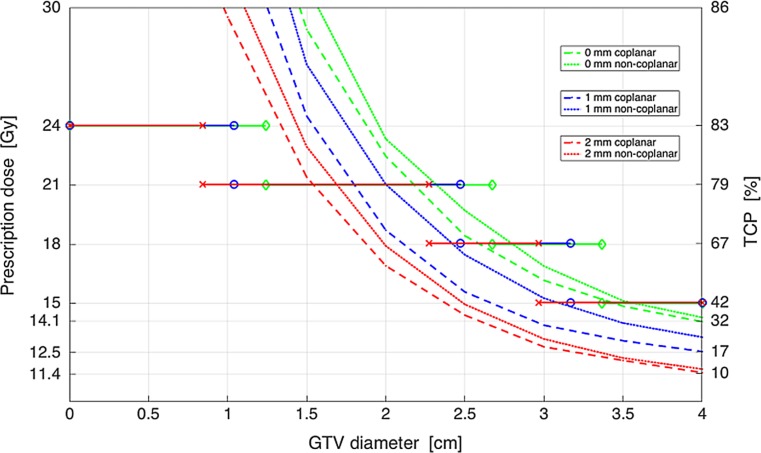
The prescribed dose and predicted TCP for single-fraction IDP with the V_12Gy_ = 10 cm^3^ constraint as a function of the GTV diameter for coplanar and non-coplanar beam arrangements with a GTV-PTV margin of 0–2 mm and spherical GTVs. Legend: *Gy* Gray, *GTV* gross tumor volume, *TCP* tumor control probability, *IDP* isotoxic dose prescription, *V*_12G__y_ volume of healthy brain tissue which is irradiated to 12 Gray or more, *PTV* planning target volume. A GTV-PTV margin enlargement results in an increase of the PTV size. An increase in the PTV size results in different cut-offs with a PTV size-based dose prescription with 24, 21, 18, and 15 Gy. The *diamond* (*green line*), *circle* (*blue line*), and *cross* (*red line*) represent the cut-offs with dose prescriptions with GTV-PTV margins of 0, 1, and 2 mm, respectively

**Fig. 4:**
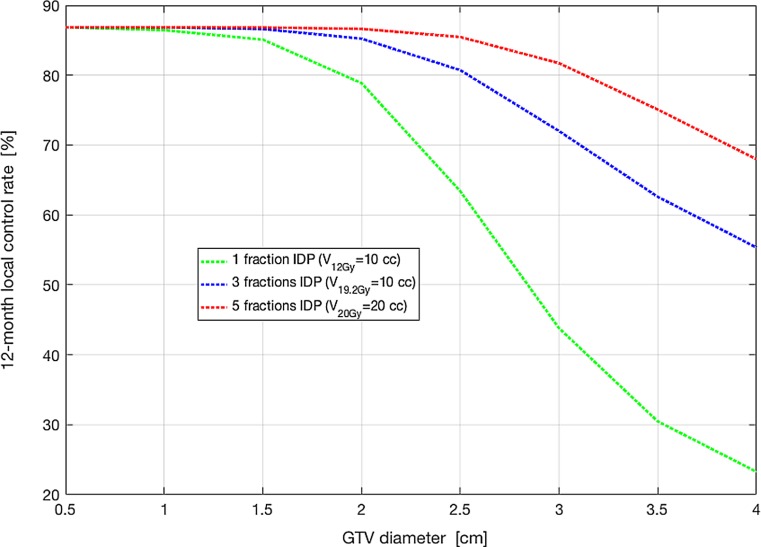
Potential of IDP to safely increase the TCP in large BM using a single, three-, and five-fraction schedule respectively. Legend: *IDP* isotoxic dose prescription, *TCP* tumor control probability, *BM* brain metastases, *V*_12Gy_ volume of healty brain tissue which is irradiated to 12 Gray or more, *V*_19.2Gy_ volume of healthy brain tissue which is irradiated to 19.2 Gray or more, *V*_20Gy_ volume of healthy brain tissue which is irradiated to 20 Gray or more. The used normal tissue constraints were a V_12Gy_ = 10 cm^3^, V_19.2_ _Gy_ = 10 cm^3^, and V_20Gy_ = 20 cm^3^ for single, three-, and five- fraction IDP respectively

As shown in Fig. 3, for a 0 mm GTV-PTV margin, single-fraction IDP with the V_12Gy_ = 10 cm^3^ constraint offers no potential for isotoxic dose escalation in spherical GTVs with diameters >2 cm, even when non-coplanar beams are used. For BM with a GTV diameter of 4 cm, this approach achieved an IDP dose of 14.1 Gy with a significantly lower TCP of 32% compared to the 42% that was predicted for the PTV size-based dose prescription at 15 Gy. Therefore, we have explored the potential of three-fraction and five-fraction IDP to increase the TCP, using a V_19.2_ _Gy_ = 10 cm^3^ and V_20Gy_ = 20 cm^3^ respectively. For BM with a 4 cm diameter, using a 1 mm GTV-PTV margin and a non-coplanar beam arrangement, the predicted TCP was increased from 23% using single fraction IDP to 55 and 68% using a three-fraction IDP and five-fraction IDP respectively (Fig. 4).

## Discussion

In this in silico study, the potential of IDP was investigated for improving the TCP from 42% with a single fraction of 15 Gy in large BM up to 4 cm diameter, while simultaneously respecting an acceptably low NTCP limit. The concept of IDP is of clinical relevance for BM with a diameter of 2 cm or more, as the constraint of a V_12Gy_ of 10 cm^3^ for the surrounding brain tissue is exceeded with SRS independent of the GTV-PTV margin and the beam arrangement with the current PTV-based dose prescription (Fig. 1). In a large cohort of patients treated with SRS alone for a maximum of 3 BM, the median diameter of the BM was 2.3 cm, so this study is of relevance for at least half of BM patients treated with SRS in daily clinical practice [2]. Despite avoiding a GTV-PTV margin and exploiting a coplanar beam arrangement, the single-fraction IDP SRS approach with the V_12Gy_ = 10 cm^3^ constraint for the nearby healthy brain tissue did not improve the predicted TCP over the standard SRS dose prescription with 15 Gy. As expected, five-fraction IDP had a better therapeutic ratio than single-fraction IDP. The predicted gain of from 32 to 73% in 1‑year TCP using five-fraction IDP instead of single-fraction IDP is significant. Such gain is especially relevant for oligometastases patients treated with curative intent in whom ablation of metastases and hence maximization of TCP is the goal. For patients with a relatively short life expectancy (for example, 6 months) treated with palliative intent, a lower 1‑year TCP could be considered acceptable. For these patients, a single-fraction approach having a relatively low 1‑year TCP may be preferred over a multiple-fraction approach for patient convenience.

To further improve the 1‑year TCP above 73%, the normal tissue constraint V_20Gy_ = 20 cm^3^ needs to be relaxed, but this may result in an unacceptably high risk of radionecrosis. Alternatively, an approach with more than five fractions could be investigated if this approach increases the therapeutic ratio. The calculated TCPs are based on the model of Wiggenraad based on single-fraction SRS data for BM, and the same model was used to calculate the TCP with five fraction SRS [4]. However, a fractionated approach may be beneficial for re-oxygenation of the tumor, which may increase its radiosensitivity [21]. Therefore, the calculated TCP of 73% in very large BM (e. g., with a diameter of 4 cm) may be an underestimation of a clinically observed TCP using five fraction IDP. The current TCP model includes only prescription dose as a prognostic variable, but further extension of this model with other factors such as BM volume and possibly imaging characteristics reflecting hypoxia may further improve its accuracy. However, the influence of tumor size is difficult to quantify because in many series, lower doses are prescribed for larger BM or prescribed doses vary widely. Furthermore, the model needs to be externally validated and calibrated in other patient cohorts treated with SRS for BM. Dose–volume thresholds for an acceptably low risk of radionecrosis for schemes other than single-fraction and five-fraction SRS do not exist in literature.

To exploit the full potential of IDP in SRS, it is necessary to minimize the GTV-PTV margin and to optimize the beam arrangements by increasing the setup accuracy (6 degrees of freedom couch and a robust frameless mask) [22, 23]. Taking into account that the risk of radionecrosis increases rapidly above 10% as the V_12Gy_ exceeds 10 cm^3^ for single-fraction SRS, it is clinically highly relevant to strive for a smaller GTV-PTV margin and to explore the feasibility of dose delivery with non-coplanar beam arrangements [14]. This is supported by a recently published randomized trial demonstrating that a decrease of the GTV-PTV margin from 3 to 1 mm does not decrease the local control probability for LINAC-based SRS [17]. As this study showed that there was a significantly larger V_12Gy_ in the 3 mm GTV-PTV margin group, the authors stated that a 1 mm GTV-PTV margin should be used to avoid any unnecessary risk of radionecrosis and serious risk of neurologic morbidity.

A limitation of our research is the lack of clinical validation of the models to predict TCP and NTCP. The TCP model is based on retrospective clinical studies [4]. Prospective clinical validation is needed for the NTCP model by using a V_12Gy_ = 10 cm^3^ constraint for single-fraction IDP, a V_19.2_ _Gy_ = 10 cm^3^ constraint for three-fraction IDP, and a V_20Gy_ = 20 cm^3^ constraint for five-fraction IDP [3, 14, 20].

In the last few years, much efforts have been made to safely increase the TCP of SRS in large BM using standard dose prescription and mild hypofractionation [24–29]. However, in all these efforts the main drawback remains that a fixed dose will always result in a variety of radionecrosis risks with different sized BM. In a palliative setting of metastasized cancer patients it seems more appropriate to make the statement of “do not harm the patient”. Therefore the usage of IDP with the principle of a low risk of radionecrosis with any sized BM is preferable over fixed SRS dose prescription.

In conclusion, using three-fraction or five-fraction IDP the predicted TCP can be increased safely to 55 and 68% respectively in BM with a diameter of 4 cm with a low risk of radionecrosis. Using IDP, the therapeutic ratio of SRS in large BM can be increased compared to current SRS dose prescription.

## Figures and Tables

**Fig. 5:**
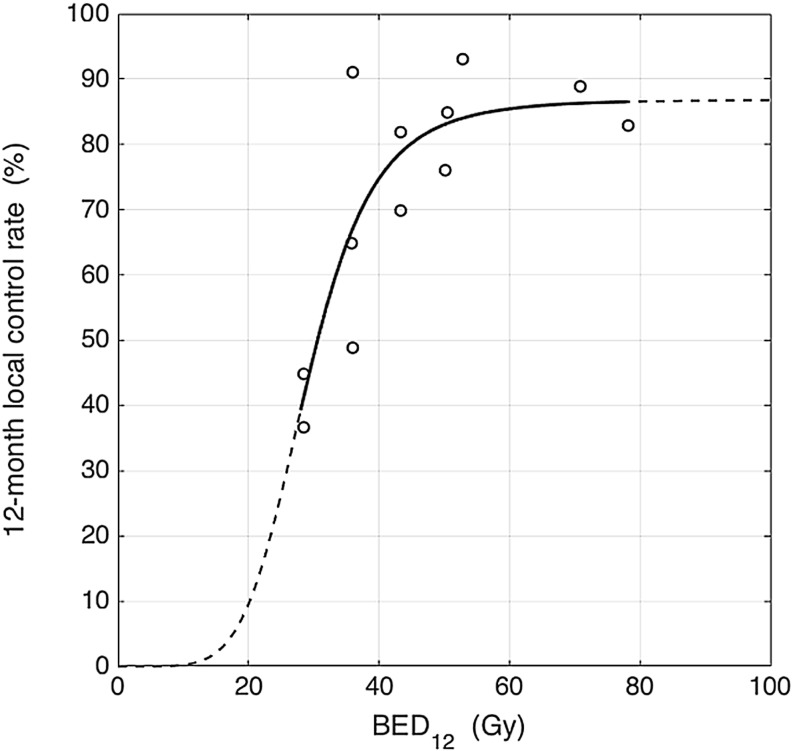
A fitted dose-response curve based on data from Wiggenraad et al. [4]

## Appendix

### Fitted TCP model for 12-month local rate

In Wiggenraad et al. [4], a dose–response relation between the biologically effective dose of the linear-quadratic-cubic model using an α/β of 12 Gy (BED_12_) and the 12-month local control rates were constructed by eye fitting. Herein we obtained a statistical better fit by first digitizing the data points from Wiggenraad’s figure [4] using GraphClick software (version 3.0.2, Arizona Software) and subsequently fitting a logistic dose–response model by the maximum likelihood estimation:

$$\textit{TCP}(D)=\frac{\textit{TCP}_{\max}}{1+\left(\frac{D_{50}}{D}\right)^{4\gamma _{50}}}$$

where *D* = BED_12_, *D*_50_ is the BED_12_ at 50% local control, *γ*_50_ is the normalized slope at *D*_50_, and *TCP*_max_ is the asymptotic local control rate for large *D*. The fitted dose–response graph is shown in Fig. 5.

The model parameters obtained are *D*_50_ = 28.97 Gy (95% confidence interval [CI]: 24.80–33.14 Gy), *γ*_50_ = 1.41 (95% CI: 0.40–2.87), and *TCP*_max_ = 86.86% (95% CI: 70.62–103.10%).

## References

[1] Bohoudi O, Bruynzeel AM, Lagerwaard FJ, Cuijpers JP, Slotman BJ, Palacios MA (2016). Isotoxic radiosurgery planning for brain metastases. Radiother Oncol.

[2] Zindler JD, Rodrigues G, Haasbeek CJ (2013). The clinical utility of prognostic scoring systems in patients with brain metastases treated with radiosurgery. Radiother Oncol.

[3] Shaw E, Scott C, Souhami L (2000). Single dose radiosurgical treatment of recurrent previously irradiated primary brain tumors and brain metastases: final report of RTOG protocol 90-05. Int J Radiat Oncol Biol Phys.

[4] Wiggenraad R, Verbeek-de Kanter A, Kal HB, Taphoorn M, Visser T, Struikmans H (2011). Dose-effect relation in stereotactic radiotherapy for brain metastases: a systematic review. Radiother Oncol.

[5] International Commission on Radiation Units and Measurements (2010). ICRU Report 83: Prescribing, recording and reporting intensity-modulated photonbeam therapy (IMRT). J Icru.

[6] https://www.clinicaltrials.gov/ct2/results?term=large+brain+metastases&Search=Search

[7] Hoffmann AL, Troost EG, Huizenga H, Kaanders JH, Bussink J (2012). Individualized dose prescription for hypofractionation in advanced non-small-cell lung cancer radiotherapy: an in silico trial. Int J Radiat Oncol Biol Phys.

[8] Beasley M, Driver D, Dobbs HJ (2005). Complications of radiotherapy: improving the therapeutic index. Cancer Imaging.

[9] van Elmpt W, Öllers M, Velders M (2008). Transition from a simple to a more advanced dose calculation algorithm for radiotherapy of non-small 5 cell lung cancer (NSCLC): implications for clinical implementation in an individualized dose-escalation protocol. Radiother Oncol.

[10] De Ruysscher D, van Baardwijk A, Steevens J (2012). Individualised isotoxic accelerated radiotherapy and chemotherapy are associated with improved long-term survival of patients with stage III NSCLC: a prospective population-based study. Radiother Oncol.

[11] Baardwijk A, Wanders S, Boersma L (2010). Mature results of an individualized radiation dose prescription study based on normal tissue constraints in stages I to III non-small-cell lung cancer. J Clin Oncol.

[12] Baardwijk A, Bosmans G, Bentzen SM (2008). Radiation dose prescription for nonsmall-cell lung cancer according to normal tissue dose constraints: an in silico clinical trial. Int J Radiat Oncol Biol Phys.

[13] Zindler JD, Thomas CR, Hahn SM, Hoffmann AL, Troost EG, Lambin P (2015). Increasing the therapeutic ratio of stereotactic ablative radiotherapy by individualized isotoxic dose prescription. J Natl Cancer Inst.

[14] Marks LB, Yorke ED, Jackson A (2010). Use of normal tissue complication probability models in the clinic. Int J Radiat Oncol Biol Phys.

[15] Lo SS, Sahgal A, Chang EL (2013). Serious complications associated with stereotactic ablative radiotherapy and strategies to mitigate the risk. Clin. Oncol..

[16] Timmerman RD (2008). An overview of hypofractionation and introduction to this issue of seminars in radiation oncology. Semin Radiat Oncol.

[17] Kirkpatrick JP, Wang Z, Sampson JH (2015). Defining the optimal planning target volume in image-guided stereotactic radiosurgery of brain metastases: results of a randomized trial. Int J Radiat Oncol Biol Phys.

[18] Fokas E, Henzel M, Surber G, Kleinert G, Hamm K, Engenhart-Cabillic R (2012). Stereotactic radiosurgery and fractionated stereotactic radiotherapy: comparison of efficacy and toxicity in 260 patients with brain metastases. J Neurooncol.

[19] Wegner RE, Leeman JE, Kabolizadeh P (2015). Fractionated stereotactic radiosurgery for large brain metastases. Am J Clin Oncol.

[20] Ernst-Stecken A, Ganslandt O, Lambrecht U, Sauer R, Grabenbauer G (2006). Phase II trial of hypofractionated stereotactic radiotherapy for brain metastases: results and toxicity. Radiother Oncol.

[21] Nahum AE (2015). The radiobiology of hypofractionation. Clin. Oncol..

[22] Seung SK, Larson DA, Galvin JM (2013). American College of Radiology (ACR) and American Society for Radiation Oncology (ASTRO) Practice Guideline for the Performance of Stereotactic Radiosurgery (SRT). Am J Clin Oncol.

[23] Seravalli E, van Haaren PM, van der Toorn PP, Hurkmans CW (2015). A comprehensive evaluation of treatment accuracy, including end-to-end tests and clinical data, applied to intracranial stereotactic radiotherapy. Radiother Oncol.

[24] Baliga S (2017). Fractionated stereotactic radiation therapy for brain metastases: a systematic review with tumour control probability modelling. Br J Radiol.

[25] Toma-Dasu I, Sandström H, Barsoum P, Dasu A (2014). To fractionate or not to fractionate? That is the question for the radiosurgery of hypoxic tumors. J Neurosurg.

[26] Jeong WJ, Park JH, Lee EJ, Kim JH, Kim CJ, Cho YH (2015). Efficacy and safety of fractionated stereotactic radiosurgery for large brain metastases. J Korean Neurosurg Soc.

[27] Ishihara T, Yamada K, Harada A, Isogai K, Tonosaki Y, Demizu Y, Miyawaki D, Yoshida K, Ejima Y, Sasaki R (2016). Hypofractionated stereotactic radiotherapy for brain metastases from lung cancer : evaluation of indications and predictors of local control. Strahlenther Onkol.

[28] Kocher M, Wittig A, Piroth MD, Treuer H, Seegenschmiedt H, Ruge M, Grosu AL, Guckenberger M (2014). Stereotactic radiosurgery for treatment of brain metastases. A report of the DEGRO working group on stereotactic radiotherapy. Strahlenther Onkol.

[29] Wiggenraad R, Verbeek-de Kanter A, Mast M, Molenaar R, Kal HB, Lycklama à Nijeholt G, Vecht C, Struikmans H (2012). Local progression and pseudo progression after single fraction or fractionated stereotactic radiotherapy for large brain metastases. A single centre study. Strahlenther Onkol.

